# Radiotheranostics in Low- and Middle-Income Countries: Challenges, Practice, and Prospects

**DOI:** 10.1055/s-0045-1812307

**Published:** 2025-10-14

**Authors:** Ismaheel O. Lawal, Honest Ndlovu, Joseph Kabunda, Kgomotso M. Mokoala, Mike M. Sathekge

**Affiliations:** 1 Department of Radiology and Imaging Sciences, Emory University, Atlanta, Georgia, United States; 2 Department of Nuclear Medicine, University of Pretoria, Pretoria, South Africa; 3 Nuclear Medicine Research Infrastructure, Steve Biko Academic Hospital, Pretoria, South Africa

**Keywords:** radiotheranostics, nuclear medicine, low- and middle-income countries, prostate cancer, ^225^ Ac-PSMA, ^177^ Lu-PSMA

## Abstract

There is a global rise in the number of new cancer diagnoses and cancer deaths. Rising new cancer diagnoses and deaths from low- and middle-income countries (LMICs) are the biggest contributors to this global trend. Efforts geared toward prevention, timely diagnosis, effective treatment, and efficient cancer survivorship programs are needed to address the rising scourge of cancer in LMICs. Radiotheranostics entails using radiopharmaceuticals for diagnostic imaging and therapy of diseases. Functional imaging, as in radiotheranostics, is more sensitive for disease detection and treatment response assessment than conventional cross-sectional imaging. Therefore, radiotheranostics has the potential to address some of the strategies to curtail the rising scourge of cancer and its mortality in LMICs, including timely diagnosis, effective management, and disease surveillance. Many key issues hinder the widespread availability, access, and utilization of nuclear medicine (NM) and radiotheranostics services in LMICs. These issues include scarcity of trained (NM) professionals, lack of training for (NM) personnel, poor infrastructure, inadequate awareness of NM and radiotheranostics, poor funding, and poorly conceived regulations that stifle NM practice. Despite these hindrances, many success stories have emerged from LMICs regarding clinical application of radiotheranostics. For example, many practice-defining studies have been published by groups from LMICs regarding prostate-specific membrane antigen-targeted imaging and therapy of prostate cancer. Specifically, notable contributions have been made to the literature by groups from South Africa, India, and Türkiye on the safety and efficacy of 225Ac-PSMA-617 for therapy of advanced prostate cancer. Through the intervention of many international organizations, governments, and private sectors, there has been a steady improvement in the awareness, availability, access, and utilization of NM and radiotheranostics services in LMICs.

## Introduction

An estimated 20 million new cancer cases were diagnosed in 2022, and 9.7 million cancer-related deaths were estimated for the same year. 1 It is projected that by the year 2050, there will be 35 million new cancer diagnoses, representing a 77% increase from the 20 million cases estimated for 2022. 1 This projected rise in the global burden of cancer will be driven by rising cancer cases in low- and middle-income countries (LMICs), with a predicted 400 and 168% rise in the number of new cancers in low-income countries and middle-income countries, respectively, compared to 53% rise for high-income countries (HICs). 2 LMICs will also be significant drivers of future global cancer mortality trends, with approximately three-quarters of all cancer deaths projected to occur in LMICs by the year 2030. The rising number of new cancer diagnoses in LMICs is due to multiple factors, including rising population, increasing life expectancy, growing urbanization, and changing lifestyle. 3 Concerted efforts geared toward prevention, timely diagnosis, effective treatment, and efficient cancer survivorship programs are needed to address the rising scourge of cancer in LMICs.

Theranostics is a contraction derived from therapy and diagnosis. Radiotheranostics entails using radiopharmaceuticals for diagnostic imaging and therapy of diseases. 4 In radiotheranostics, radiopharmaceuticals are designed to bind molecular targets expressed by the cancer cells for imaging and therapy. Imaging serves to confirm the in vivo expression of the molecular target of interest, which is exploited by a therapeutic radiopharmaceutical for targeted delivery of cytotoxic radiation to tumor cells. Through this mechanism, radiotheranostics is a personalized therapy that ensures that only patients whose disease expresses the molecular targets of interest and have the highest chances of response are treated with a given radiopharmaceutical while preventing futile treatment in patients with lower odds of response. 5 Moreover, molecular imaging with radiopharmaceuticals is more sensitive for disease detection and response assessment than conventional cross-sectional imaging, thereby contributing to improved staging and re-staging of disease. The ability of molecular imaging to detect response or otherwise early during treatment helps identify effective treatment that should be continued versus ineffective treatment that should be discontinued. 6 Therefore, radiotheranostics has the potential to address some of the strategies to curtail the rising scourge of cancer and its mortality in LMICs, including timely diagnosis, effective management, and disease surveillance. This review aims to briefly highlight the key challenges militating against the full-scale deployment of radiotheranostics as an effective tool for cancer diagnosis and treatment in LMICs ( Table 1 ). In the second section of this article, we will discuss the reported success of radiotheranostics deployment in LMICs in oncology using targeted radionuclide therapy of metastatic castrate-resistant prostate cancer as a case study. In the end, we will highlight the current efforts to improve the availability and utilization of nuclear medicine and radiotheranostics services in LMICs.

**Table 1:** Radiotheranostics in LMICs: challenges, current efforts, and future directions

Challenge	Current efforts	Future steps
Workforce shortage	- IAEA-sponsored training programs and fellowships - Regional workshops and technical cooperation projects - Limited local training initiatives in select LMICs	- Establish accredited nuclear medicine training centers in LMICs - Incentivize retention through career development and local research opportunities - Expand virtual and hybrid training platforms
Infrastructure deficit	- Installation of PET and cyclotron facilities in select LMICs (e.g., Nigeria, Ghana) - Support from international donors and the private sector - IAEA's IMAGINE database for resource mapping	- Develop regional imaging hubs with shared access - Promote public–private partnerships for equipment procurement and maintenance - Implement scalable, modular facility designs for cost efficiency
Radiopharmaceutical supply chain	- Reliance on single vendors for 99mTc generators - Regional production in South Africa and India - IAEA coordination on isotope availability	- Establish regional radiopharmaceutical production centers - Diversify supply chains and vendors - Invest in compact cyclotrons and generator technologies tailored for LMIC settings
Limited utilization	- Awareness campaigns by SNMMI, WARMTH, and IAEA - Pilot programs introducing PSMA therapy in LMICs - Local guidelines published in Iran, South Africa, etc.	- Integrate radiotheranostics into national cancer control plans - Educate referring clinicians to increase appropriate referrals - Expand insurance coverage and government subsidies for nuclear medicine
Affordability and funding	- Out-of-pocket payments dominate - Some donor-funded treatments and trials - Limited universal health coverage	- Advocate for inclusion in essential health benefit packages - Explore tiered pricing and local manufacturing - Mobilize global health financing mechanisms (e.g., GAVI-like models for radiotheranostics)
Regulatory barriers	- IAEA support for regulatory framework development - Some LMICs have overly restrictive radionuclide use laws (e.g., armed escorts for isotopes)	- Train regulators in radiation safety and nuclear medicine - Harmonize regulations across regions - Develop LMIC-specific guidelines balancing safety and feasibility
Exclusion from clinical trials	- Limited LMIC participation in registries and trials - Some multicenter studies include LMICs (e.g., WARMTH Act study)	- Mandate LMIC inclusion in global trials - Build local trial infrastructure and ethics boards - Encourage pharma partnerships with LMIC research institutions

Abbreviations: IAEA, International Atomic Energy Agency; LMICs, low- and middle-income countries; SNMMI, Society of Nuclear Medicine and Molecular Imaging; WARMTH, World Association of Radiopharmaceutical and Molecular Therapy.

## The Key Challenges to Radiotheranostic Practice in LMICs

The successful practice of theranostics and its integration as a standard-of-care treatment strategy requires many factors, including workforce and training, awareness among referring healthcare providers, and resources in the form of equipment, radiopharmaceutical supply, and payment for the care. 7 The recent development of novel radiotheranostic agents and their approval by regulatory authorities in different jurisdictions worldwide has led to a sharp rise in global awareness regarding their efficacy and utilization in the routine care of cancer patients. This rise in awareness and utilization of radiotheranostics has yet to be replicated uniformly across the LMICs. This section will discuss key factors militating against the availability, access, and integration of radiotheranostics into the cancer therapy armamentarium in LMICs.

### Workforce and Training

The practice and advancement of radiotheranostics require the expertise of different groups of professionals, including nuclear medicine physicians, clinician scientists, radiochemists, radiopharmacists, medical physicists, nuclear medicine technologists, and nursing staff. The report of a recent survey of the global nuclear medicine workforce conducted as part of the Lancet Oncology Commission on Radiotherapy and Theranostics reveals a grim picture of the worldwide shortage of all categories of professionals with the requisite training and expertise for the rising global needs of theranostics. 8 While this shortage is global in scale, there is a significant disparity in the nuclear medicine staffing capacity between the HICs and LMICs. For example, the median number of nuclear medicine physicians per million inhabitants is 7.0, 1.2, 0.2, and 0.0 for HICs, upper-middle-income, lower-middle-income, and low-income countries, respectively. There is a similar global shortage with a significant disparity in the staffing of other categories of the nuclear medicine workforce. The median numbers of nuclear medicine technologists per million inhabitants for HICs, upper-middle-income, lower-middle-income, and low-income countries are 6.7, 0.9, 0.1, and 0.0, respectively. 8

Judging by the trajectory of the growth of radiotheranostics, 9 the global shortage of the nuclear medicine workforce will likely continue to worsen into the near future. An increasing number of clinical trials are ongoing, which may lead to the approval of novel radiotheranostic agents or expand the indications for the approved agents. 4 For example, the recently reported favorable results of the NETTER-2 trial may soon bring lutetium-177 DOTATATE to the frontline, making it a first-line agent for treating metastatic or inoperable differentiated neuroendocrine tumors, the first radiotheranostic agent to attain a first-line status. 10 In a recent estimation, 41,500 patients in the United States alone are expected to require 150,000 treatment cycles of lutetium-177-based radiotheranostics for neuroendocrine tumors and metastatic prostate cancer. About 140 theranostic centers administering four treatment cycles per workday would be necessary to treat these patients. This shows that, even in HICs, there is an urgent need to ramp up capacity to deliver radiotheranostics. Therefore, there is a potential for an increasing rate of workforce migration from LMICs to HICs due to economic reasons.

Traditionally, nuclear medicine has been primarily a diagnostic specialty. With advances in radiotheranostics, therapy is expected to constitute about 60% of all nuclear medicine clinical services. 9 As nuclear medicine transforms from a primarily diagnostic to a therapy-driven specialty, there is a need to train and retrain current crops of nuclear medicine practitioners to build capacity for therapy delivery. 11 The current shortage in the workforce, which is expected to worsen in the near future, also requires the training of a new crop of professionals needed to deliver radiotheranostics. Unfortunately, training new professionals for radiotheranostics is not a quick fix, considering it requires many years of training to attain the competence necessary for independent theranostic practice. Also, formal training in the different nuclear medicine specialties is not available in most LMICs. The International Atomic Energy Agency (IAEA) has played a pivotal role in the past decades in facilitating and sponsoring overseas training for professionals in the LMICs to build capacity for successful nuclear medicine practice in member states across LMICs. 7 12 Through its regular technical programs and conferences, the IAEA also provides training opportunities for nuclear medicine professionals in the LMICs. 13

### Infrastructure

The cost of establishing a nuclear medicine service is enormous. Nuclear medicine services require special building designs to minimize radiation exposure. Highly specialized imaging, radiation detection, and radiation protection equipment are also required, which are costly to procure. Regular maintenance is required to keep these highly specialized pieces of equipment in optimal functioning mode. The exorbitant cost of procuring and maintaining nuclear medicine equipment may hinder its availability in LMICs. The integral role that imaging plays in theranostics, specifically, and cancer care in general, indicates that the shortage of nuclear medicine equipment in LMICs is an important contribution to the limited availability of radiotheranostics in these countries. According to the IAEA IMAGINE database, there are 3.5, 0.3, 0.1, and 0.0 PET scanners per million inhabitants in the high-income, upper-middle-income, low-middle-income, and low-income countries, respectively. 14 The respective numbers for the installed SPECT scanners are 17.6, 1.6, 0.3, and 0.0. 14

The substantial cost of scale-up of medical imaging, including radionuclide imaging for theranostics, is well known. Because of this, some argue that investment in advanced imaging technologies like SPECT and PET is not a priority for LMICs. This is a misconception, as robust data show significant returns from investment in cancer imaging scale-up. 15 For example, the return on investment on scale-up of treatment and quality of care without imaging scale-up is $6.15 per dollar invested compared to a return of $12.43 per dollar invested in the combined scale-up of treatment, quality of care, and imaging in cancer care. 15 Scale-up of cancer imaging infrastructure also saves lives across different cancers, as comprehensively documented in the recently published report of the Lancet Oncology Commission. 15 Data from the prospective oncologic PET registry study from the United States also show substantial management changes in 37% of patients across various cancers due to PET imaging findings. 16 These indicate that investment in advanced imaging techniques like PET saves the cost and lives of cancer patients.

### Radiopharmaceutical Supply

Radiopharmaceuticals are required for imaging and therapy in radiotheranostics. Therefore, a robust radiopharmaceutical supply chain is requisite for a successful radiotheranostic program. Large-size unit reactors for producing diagnostic and therapeutic radionuclides are present in a handful of countries, including Australia, Belgium, the Netherlands, and South Africa. 17 Worldwide distribution of installed cyclotrons producing medical radioisotopes shows a disproportional concentration in HICs relative to LMICs. For example, approximately 335 and 215 cyclotrons are installed in the United States and Japan compared to approximately 25 in Africa. 17 Technetium-99m ( ^99m^ Tc) obtained from the ^99m^ Tc generators from the decay of the parent molybdenum-99 is the workhorse of general nuclear medicine. A recent survey of the global supply chain of radiopharmaceuticals reported 32 suppliers of ^99m^ Tc generators worldwide, with 18 supplying a single country. 18 The United States consumes 50% of the global ^99m^ Tc generator production. Supply to Africa is most constrained, with many countries relying on supply from a single vendor. 18 Dependence on a single vendor for the supply of a product that is already in short supply globally demonstrates the fragility of the global supply chain of radiopharmaceuticals, with LMICs bearing the brunt of this problem.

The worldwide distribution of therapy radionuclides also faces regular disruption due to the fragility of their supply chain. The current supply challenges are likely to worsen, considering the projected rapid growth of radiotheranostics and the rapidly aging available infrastructures for radionuclide production. 19

### Utilization of Radiotheranostics

Radiotheranostics began with the use of radioactive iodine therapy for thyroid disease treatment about 80 years ago. 20 21 Since then, several other theranostic agents have been developed and approved for treating various malignant disorders. 9 By far, the most rapid growth of radiotheranostics occurred in the last decade with the approval of ^177^ Lu-DOTATATE and ^177^ Lu-PSMA for the treatment of differentiated metastatic/inoperable neuroendocrine tumors and metastatic castration-resistant prostate cancer, respectively. This rapid growth in radiotheranostics utilization has not been uniformly replicated across the LMICs. 22 Compared to the HICs, radiotheranostics utilization in LMICs is generally limited, even for the more affordable established radiotheranostics agents, such as radioactive iodine, which have been used for many decades. 23 For example, radioactive iodine utilization for hyperthyroidism and thyroid cancer treatment is 161.9 per million population in the HICs versus 125.0, 31.3, and 0.3 per million population in the upper-middle-income, lower-middle-income, and low-income countries, respectively. 23 The trend is similar for radiotheranostics utilization for bone pain treatment in patients with bone metastases, with a utilization per million population of 37.5 for HICs versus 5.3, 0.5, and 0.0 for upper-middle-income, lower-middle-income, and low-income countries, respectively. 23

Several factors, including those discussed earlier, contribute to the limited availability of radiotheranostics services and their underutilization in LMICs. Unfortunately, the limited available radiotheranostics are also underutilized. Several factors contribute to this, including the low volume of referrals and the cost of service.

Due to its limited availability, the knowledge regarding radiotheranostics among the referring clinicians may be limited, hence low referral of deserving patients to nuclear medicine for radionuclide therapy. 24 Frequent disruptions to radiotheranostics services arising from a fragile radiopharmaceutical supply chain and frequent imaging equipment downtime resulting from limited technical support may, over time, result in referral apathy in the referring clinicians toward radiotheranostics. 25

Affordability is another driver of the limited utilization of radiotheranostics as an oncology treatment. There is limited universal health coverage provision by the government or comprehensive health insurance with cancer care coverage in LMICs. Hence, patients pay out of pocket in many countries for medical care. As many LMICs depend on radiopharmaceutical importation from other countries, the cost of radiotheranostics is often high, beyond the reach of the average population. Therefore, many deserving patients cannot access radiotheranostics care due to affordability issues, even when available. 25 26

### Regulatory Barrier

The practice of radiotheranostics is highly regulated to standardize practice and ensure the safety of patients, staff, and the public. 23 Various aspects of nuclear medicine and radiotheranostics that are strictly regulated for standardization and safety include radiopharmaceutical production, transport of radioactive substances, design of the facility where radioactive materials are used, the cameras and other radiation detection devices, radiopharmaceutical administration, and image acquisition and interpretation. 27 The technical know-how required to enact enabling regulations is lacking in many LMICs. In many instances, regulatory staff with limited knowledge of radiation science and radiation safety make laws to regulate radiotheranostic practice in some LMICs. Due to the general phobia for radiation, these laws are usually stricter than necessary, consequently stifling the practice of nuclear medicine and radiotheranostics rather than ensuring its safe practice. For example, due to the limited knowledge regarding radionuclides used in nuclear medicine and radiotheranostics, some LMICs require armed police escorts to transport radionuclides for medical use. This stifling regulation arises from the misconception that medical isotopes can be hijacked by criminals to make dirty bombs.

## The Practice of Radiotheranostics in LMICs—The Success Stories

Despite the significant challenges hindering the practice and growth of radiotheranostics in the LMICs, there are many successes in the use of radionuclides for imaging and therapy coming out of some LMICs. In this section, we will discuss the rich literature published by researchers from LMICs regarding the use of radiotheranostics in prostate cancer treatment. Radioligands targeting prostate-specific membrane antigen (PSMA) for imaging and therapy of prostate cancer are the latest approved radiotheranostics agents. These PSMA-targeting radioligands have changed the fortune of nuclear medicine due to the sheer number of patients who can benefit from imaging and treatment with them. Some of the early and pivotal works shaping the clinical use of PSMA radioligand therapy (PRLT) were done by researchers in the LMICs alone or in collaboration with researchers in HICs.

### SPECT and PET Imaging of Prostate Cancer in the LMICs

Imaging is an integral component of radiotheranostics as it is required for detecting the presence of disease, quantifying disease extent, determining target expression, determining dose to the tumor and normal organs (dosimetry), evaluating response to treatment, and detecting disease recurrence. PSMA, a transmembrane glycoprotein, is the most successful molecular target for imaging and therapy of clinically significant prostate cancer. 28 It is overexpressed in prostate cancer compared to normal prostate tissue or benign prostate disorders, and its level of expression is proportional to the level of disease aggressiveness. 29 30 Several small-molecule ligands targeting prostate cancer-expressed PSMA have been labeled with PET radionuclides, such as gallium-68 ( ^68^ Ga) and fluorine-18 ( ^18^ F), for PET imaging of prostate cancer in different clinical settings. The most successful PSMA-targeting PET radiopharmaceuticals are ^68^ Ga-PSMA-11, ^18^ F-DCFPyL, ^18^ F-PSMA-1007, ^68^ Ga-PSMA-I&T, and ^18^ F-rhPSMA-7.3. 31 32 33 34 35 36 ^68^ Ga-PSMA-11, ^18^ F-DCFPyL, and ^18^ F-rhPSMA-7.3 are approved by most regulatory authorities worldwide, including the United States Food and Drug Administration (FDA). These approvals were sequel to favorable results of phase 3 clinical trials demonstrating their excellent diagnostic performance or superiority over conventional imaging modalities for prostate cancer imaging. 31 32 33 35 36 37 38

The registry trials for the PSMA radiopharmaceuticals recruited patients from HICs in North America, Europe, and Australia. However, several studies shaping the diagnostic and prognostic utility of PSMA PET imaging of prostatic cancer have been conducted in LMICs. Given the multiplicity of PSMA agents for PET imaging of prostate cancer, it is important to understand the nuanced differences in their diagnostic performance. Studies from the University of Pretoria in South Africa have compared the diagnostic performance of different PSMA radioligands for staging and re-staging of prostate cancer. 39 40 For example, in a prospective study done in collaboration with researchers from Germany, the Pretoria team reported similar lesion detection rates for ^18^ F-DCFPyL and ^18^ F-PSMA-1007 in patients with newly diagnosed prostate cancer. 40 These results are intriguing, considering the low urinary excretion of ^18^ F-PSMA-1007, which may enhance local intraprostate lesion detection. Technetium-99m methylene diphosphonate ( ^99m^ Tc-MDP) bone scintigraphy is the most common nuclear medicine imaging modality done for prostate cancer imaging in LMICs due to the more widespread availability of SPECT than PET facilities. 14 With the clinical introduction of PSMA PET imaging, several groups from the LMICs have reported the superiority of PSMA PET imaging over SPECT bone scintigraphy. 41 42 43 44 45 46 These results from India, South Africa, Turkey, and other LMICs, as well as similar studies from the HICs, are driving the shift in clinical practice from bone scintigraphy toward PSMA PET imaging where available. 41 42 43 44 45 46

To improve sampling yield, prostate biopsy for histopathological diagnosis is done under imaging guidance, ultrasound or magnetic resonance imaging (MRI). Imaging is also necessary for prebiopsy differentiation of clinically significant prostate cancer (csPCa), warranting active treatment versus nonsignificant prostate cancer (Gleason score ≤6), typically treated with watchful waiting or active surveillance. Studies from India and China have contributed to the emerging body of literature demonstrating the utility of PSMA PET/CT to guide biopsy for improved detection of csPCa. 47 48 49 In the study by Zhang et al, PSMA PET-guided prostate biopsy had a higher yield for csPCa than transrectal ultrasound-guided biopsy. 48 PSMA expression level is proportional to tumor aggressiveness. Therefore, this suggests that csPCa will demonstrate higher PSMA radiotracer uptake compared to nonsignificant indolent prostate cancer. 50

Racial differences in the genetic markers driving prostate cancer have been identified, with certain genetic markers predominantly or exclusively seen in patients of African ancestry. 51 This suggests important differences between prostate cancer seen in different racial groups. Studies from South Africa leveraging the racial diversity of South Africa have explored the utility of PSMA PET imaging to interrogate the racial differences in tumor phenotype at staging and restaging of prostate cancer. 52 53 While subtle differences exist in the prostate cancer seen in White versus Black South Africans, especially at the time of initial disease diagnosis, the implication of these differences in response to treatment in general and response to PSMA radioligand therapy remains unexplored currently.

Despite the excellent diagnostic and prognostic performance of PSMA PET in prostate cancer management, many nuclear medicine centers in LMICs do not have a PET system installed. This has been a major drive for the high research interest in investigating the ^99m^ Tc-labeled PSMA radiopharmaceuticals for SPECT imaging of prostate cancer. 41 54 55 56 In one of the first studies comparing ^68^ Ga-PSMA-11 PET/CT to ^99m^ Tc-HYNIC-PSMA SPECT/CT, our group in Pretoria, South Africa, reported a high detection rate for ^99m^ Tc-HYNIC-PSMA SPECT/CT, though with a lower performance than ^68^ Ga-PSMA-11 PET/CT. 57 One reason for the lower sensitivity of SPECT imaging is its limited sensitivity for small lesions. Many other authors across different LMICs have investigated ^99m^ Tc-PSMA SPECT/CT for prostate cancer imaging, highlighting its superiority to conventional imaging with whole-body CT and ^99m^ Tc-MDP bone scintigraphy. Also, the performance of ^99m^ Tc-PSMA SPECT/CT is sufficient to identify patients for PSMA-targeted radioligand therapy, an important prerequisite for prostate cancer radiotheranostics.

### ^177^ Lu-PSMA Radioligand Therapy of Prostate Cancer in LMICs

^177^ Lu-PSMA-617 was approved for treating metastatic castration-resistant prostate cancer (mCRPC) in men previously treated with at least one taxane-based chemotherapy and at least one line of novel androgen receptor pathway inhibitors (ARPI) following favorable results from the VISION trial. 58 Due to its tolerability and efficacy, PSMA radioligand therapy (PRLT) with ^177^ Lu-PSMA-617 is a highly preferred treatment option by oncologists and patients with mCRPC worldwide. While there is a rising global demand for ^177^ Lu-PSMA-617 and a consequent ramping up of its supply, PRLT utilization in LMICs is low and highly heterogeneous. 23 Despite this, many groups have reported their experience with ^177^ Lu-PSMA-617 radioligand therapy of mCRPC across LMICs. 59 60 61 62 Interestingly, some of the earliest works reporting the safety and efficacy of upfront PRLT in hormone-sensitive prostate cancer and chemotherapy-naive mCRPC treatment were done by researchers from LMICs. 63 64 The recently published results of the phase III PSMAfore trial have confirmed the safety and efficacy of ^177^ Lu-PSMA-617 in chemotherapy-naive mCRPC patients who experienced disease progression on ARPI. 65 A recently published multicenter Turkish study is particularly deserving of commendation because of the collaborative effort that went into the reporting of the safety and efficacy of ^177^ Lu-PSMA-617 in 165 men with mCRPC. 66 The study reported a PSA response rate of 50.6% with generally tolerable side effects. Interestingly, the patients who received PRLT combined with ARPI had longer overall survival than those who received PRLT alone. 66

Some studies published from LMICs are unique in their approach, reporting the challenges associated with the use of this novel therapy in resource-constrained settings. 67 To address the peculiarities of radiotheranostics practice in resource-constrained settings, some LMICs, including Iran and South Africa, have published practice guidelines to aid practitioners in selecting patients and safely administering PSMA radioligand treatment within the context of the prevailing circumstances in the respective countries. 68 69

### ^225^ Ac-PSMA Radioligand Therapy of Prostate Cancer in LMICs

Despite its efficacy, up to 54% of mCRPC patients treated with ^177^ Lu-PSMA-617 may not achieve a response, measured by a ≥50% decline in serum prostate-specific antigen (PSA) level. 58 Also, patients who respond eventually experience disease progression, warranting further treatment. 70 PSMA-labelled to the alpha particle-emitting actinium-225 ( ^225^ Ac) is effective for mCRPC resistant or refractory to beta particle-emitting ^177^ Lu in ^177^ Lu-PSMA-617. 70 71 Unlike the beta particles of ^177^ Lu-PSMA-617, the alpha particles emitted by ^225^ Ac-PSMA-617 are highly energetic, causing irreparable double-stranded DNA and cell death without the requirement for oxygen. Due to the complicated modes of ^225^ Ac production that limit its supply considerably, 72 the global application of ^225^ Ac-PSMA-617 is currently highly limited to a few countries. 73 Of these few countries, at least three LMICs (South Africa, India, and Türkiye) have made significant contributions to the literature on the clinical applications of ^225^ Ac-PSMA-617 in mCRPC treatment.

The first contribution to the ^225^ Ac-PSMA-617 literature from an LMIC was from South Africa when the Pretoria group reported their experience with ^225^ Ac-PSMA-617 in chemotherapy-naive patients with mCRPC. PSA response was reported in 88%, and there was complete disease resolution on ^68^ Ga-PSMA-11 PET/CT in 65% of patients. 74 Following these remarkable results, the group also investigated the safety and efficacy of ^225^ Ac-PSMA-617 in other groups of patients who may typically not be qualified to receive this treatment in HICs. 75 76 For example, in a group of 53 mCRPC patients who had only been treated with androgen deprivation therapy before receiving ^225^ Ac-PSMA-617, the group reported PSA response in 91% of patients. 75 The median progression-free survival (PFS) was 4 months for patients who did not achieve PSA response versus 22 months for patients who did, highlighting the prognostic significance of attaining a PSA decline of 50% or more. The group investigated ^225^ Ac-PSMA-617 use even in an earlier setting in the disease course of advanced prostate cancer. In de novo metastatic hormone-sensitive prostate cancer, Sathekge and colleagues reported a PSA response rate of 86%, including 19% of patients who had undetectable serum PSA levels after treatment. 76 The optimum sequencing of the approved therapy agents for mCRPC remains unknown. However, ^225^ Ac-PSMA-617 is typically administered as a last-line treatment in patients who have exhausted or are ineligible for approved treatments such as taxane-based chemotherapy, ARPI, and ^177^ Lu-PSMA-617. In the LMICs, these approved agents may not be readily available to patients who need them due to resource constraints. ^225^ Ac-PSMA-617 was used earlier in the disease course in these patients due to their lack of access to the approved patients. Therefore, this limitation to practice in an LMIC serves to enrich our knowledge regarding the efficacy of ^225^ Ac-PSMA-617 in earlier settings.

The Pretoria group has also reported the safety and efficacy of ^225^ Ac-PSMA-617 use as a last-line therapy for mCRPC. 77 78 The group investigated the predictors of response to ^225^ Ac-PSMA-617 among mCRPC patients who have received varying lines of approved therapy. Among 73 men with mCRPC who were treated with 210 cycles of ^225^ Ac-PSMA-617, PSA response was achieved in 70% of them with a median PFS and overall survival (OS) of 15.2 months (95% CI: 13.1–17.4) and 18 months (95% CI: 16.2–19.9), respectively. PSA response was a significant predictor of PFS and OS, while prior treatment with ^177^ Lu-PSMA-617 was significantly associated with poorer PFS. 77 The bone is a common site of prostate cancer metastasis, and some patients may present with diffuse osseous metastasis in a superscan pattern. 79 Patients with superscan pattern of osseous metastases were excluded from the VISION trial due to the potential of bone marrow toxicity that the long range of beta particles emitted by ^177^ Lu may cause. Alpha particles have a much shorter range in tissue, limiting the radiation dose to normal tissue contiguous to the tumor focus. The Pretorial group, therefore, investigated the hematologic toxicity due to ^225^ Ac-PSMA-617 in mCRPC patients with extensive (>20 bone lesions) or superscan pattern of skeletal metastases. 78 The group reported a low incidence of severe hematologic toxicities (grade ≥3) among 106 patients who received up to nine cycles of ^225^ Ac-PSMA-617, 92.5% of whom had abnormal baseline hematologic parameters due to their disease or prior treatment they received. 78 Specifically, one patient had grade 4 thrombocytopenia, while grade 3 anemia, leukopenia, and thrombocytopenia were seen in one (0.9%), three (2.8%), and two (1.9%) patients, respectively.

Researchers from India have also made remarkable contributions to the literature on mCRPC treatment with ^225^ Ac-PSMA-617. 80 81 82 83 Xerostomia is a common treatment-related toxicity seen in almost all patients treated with ^225^ Ac-PSMA-617. 73 84 Owing to the significant impact xerostomia may have on the quality of life, there is a concern that treatment with ^225^ Ac-PSMA-617 may significantly reduce the quality of life despite a favorable disease response to treatment. Satapathy and colleagues from Chandigarh in India investigated the quality-of-life outcomes of patients treated with ^225^ Ac-PSMA-617 for mCRPC. 83 The authors reported significantly improved emotional and physical aspects of disease-related symptom domains of the validated FPSI quality-of-life questionnaire without notable deterioration in the treatment-related side effects or functional well-being domains. 83 While these favorable results were reported from a modest study population with limited follow-up duration, it is reassuring, encouraging the continued use of ^225^ Ac-PSMA-617, especially earlier in the disease course, while further evidence supporting its positive impact on quality of life is still being investigated.

A group from Istanbul in Türkiye has recently reported a Turkish experience with ^225^ Ac-PSMA-617 radioligand therapy of mCRPC. In a group of 23 patients with mCRPC refractory to ^177^ Lu-PSMA-617 and treated with one to four cycles of ^225^ Ac-PSMA-617, 50% of patients achieved disease control assessed by ^68^ Ga-PSMA PET/CT. 85 These results are encouraging, considering that these patients have no approved therapy available to them, having exhausted the approved agents, including ^177^ Lu-PSMA-617.

Researchers in the LMICs have also collaborated with groups from HICs in performing multicenter studies to provide stronger proof of the safety and efficacy of ^225^ Ac-PSMA-617 in mCRPC treatment. This collaborative effort is best exemplified by the recent multicenter WARMTH Act study, where three centers in India and one from South Africa collaborated with groups from Germany and Australia to jointly publish the largest data so far on the safety and efficacy of ^225^ Ac-PSMA-617 in heavily pretreated mCRPC patients. 86 The study included data from 488 patients treated with 1174 cycles of ^225^ Ac-PSMA-617, 66, 21, 39, 39, 32, and 2% of whom had been previously treated with docetaxel, cabazitaxel, abiraterone, enzalutamide, ^177^ Lu-PSMA-617, and radium-223 dichloride, respectively. PSA response rate was 57% with a median PFS and OS of 7.9 months (95% CI: 6.8–8.9) and 15·5 months (95% CI: 13.4–18.3), respectively. For the first time, the study reported the presence of peritoneal metastasis as a predictor of unfavorable disease outcomes (PFS and OS). Peritoneal metastasis is generally rare among patients with mCRPC. However, significant cases of peritoneal metastases were seen due to the large study population. The lesions appear as bulky peritoneal nodules in patients with a large whole-body disease burden, and the large volume of disease is probably responsible for the limited response of targeted alpha therapy in this patient subpopulation.

## Conclusion

The availability of radiotheranostics services is generally limited in LMICs. Radiotheranostics is increasingly playing a frontline role in cancer care. 87 Therefore, concerted efforts must be made to address all the challenges that hinder the availability and utilization of radiotheranostics services in LIMCs. The IAEA, through its mission of promoting the safe, secure, and peaceful uses of nuclear science and technology in its member states, has assisted several LMICs to help them build sustainable capacities in radionuclide use for imaging and therapy. The key IAEA interventions have been in the areas of personnel training and retraining, provision of imaging and non-imaging equipment, development of self-assessment tools for nuclear medicine facilities to benchmark their practice against the international best practice through IAEA's Quality Management Audits in Nuclear Medicine (QUANUM), and coordination of multicenter research activities that bring researchers from LMICs and HICs to work together to facilitate the transfer of research expertise. 7 These IAEA's interventions have helped improve access to and utilization of nuclear medicine and radiotheranostics services in LMICs. Despite the progress made, more efforts are still needed to attain universal access to nuclear medicine and radiotheranostics services in LMICs.

Other international organizations are also contributing to creating awareness and improving access and utilization of nuclear medicine and radiotheranostics services in the LMICs. In May 2023, the World Association of Radiopharmaceutical and Molecular Therapy (WARMTH) held its 18th International Conference on Radiopharmaceutical Therapy (ICRT) in Accra, Ghana, as a way of improving awareness of radiotheranostics, specifically in West Africa and Sub-Saharan Africa in general. The first PSMA radioligand therapy ever administered in Ghana was performed as part of the event. A post-congress meeting was held in Kumasi, Ghana, where WARMTH initiated nuclear medicine diagnosis and therapeutic services at the HopeXchange Medical Center in Kumasi, a modern, fully digitized center of excellence that has received tremendous support from the Medical University of Innsbruck and the Tirol-Kliniken, Austria. The Society of Nuclear Medicine and Molecular Imaging (SNMMI) has also supported nuclear medicine and radiotheranostics in LMICs in many ways. For example, in May 2023, an SNMMI expert delegation visited the Korle Bu Teaching Hospital in Ghana to help establish the country's first nuclear cardiology service. 88

There is an increasing investment in establishing new nuclear medicine and radiotheranostics centers by governments and private sectors in LMICs. For example, in the last few years, many countries in Sub-Saharan Africa (including Nigeria, Ghana, Kenya, and Tanzania) installed their first-ever PET and medical cyclotron facilities, broadening access to nuclear medicine services in the region. Similar growth is being witnessed in LMICs in other regions of the world.

The registry trials that led to the recent approval of the different radiopharmaceuticals/radioligands for nuclear medicine imaging and therapy were performed in HICs in North America and Europe. There is a need to diversify the study population recruited into future registry studies by including subjects in LMICs. This will serve many purposes, including providing data demonstrating the safety and efficacy of the investigational product in subjects from LMICs and an opportunity to transfer trial skills to researchers across LMICs. The inclusion of subjects from LMICs provides the genetic diversity needed for a comprehensive test of an investigational product, especially for a disease like prostate cancer with high inherent racial differences in the genetic markers driving the disease.
